# On the Salicylate of Sodium

**Published:** 1876-12

**Authors:** 


### ON THE SALICYLATE OF SODIUM.

In reference to this salt, Dr. H. P. V. Petershansen writes, in the Detroit *Review of Medical Pharmacy:*—

When the salicylic acid was introduced to the profession as an internal remedy, it was recommended to give it in the form of powder dispensed in wafers. But nausea was oftener noticed after this medication, wherefore compressed tablets were prapared from the acid, and given with better results. Some advise to prescribe it with two or more parts of sugar, and order to wet each powder, before it is taken, with some water. As the sodium salicylate insures the same antipyretic effect as the acid, but does not irritate the mucous membrane of the stomach and intestines, nor act in an unpleasant manner upon the brain (like quinine), or cause nausea, it is preferred now before the acid by many physicians. It may be given even in a concentrated solution(1.30). In case the salt can not be had, it may be prepared extemporaneously by solving the acid in a solution of some sodium salt (phosphate, borate, etc.).

FOR INTERNAL USE.

| | | | | |
|---|---|---|---|---|
| ℞. | Sodæ carb., | | ʒij | |
| | Aquæ font., | | ℥ijss | |
| | Acid. salicyl., | | ʒij | M. |
| ℞. | Sodæ phosph., | | ʒiv | |
| | Aquæ font., | | ℥ijss | |
| | Acid. salicyl., | | ʒij. | M. |

FOR A GARGLE.

| | | | | |
|---|---|---|---|---|
| ℞. | Acid. salicyl., | | gr.xx | |
| | Alcohol, | q. s. ad. | solut. | |
| | Aquæ font., | | ℥iv. | M. |

A part of the latter may be replaced by glycerine.

OINTMENT.

| | | | |
|---|---|---|---|
| ℞. | Acid. salicyl., | ʒj | |
| | Ungu., | ℥j | M. |

Make an ointment. A liniment of olive oil may also be advantageous in some cases.

Resuming the principal facts in regard to salicylic acid, we find that—

First. It is an excellent antiseptic when applied externally, and may, in surgery, take the place of carbolic acid on many occasions.

Second. It is, perhaps, the best antipyretic now known, quinine not excluded, when appied internally.

Third. As an internal remedy, it has no antiseptic properties.

Fourth. The best form in which it is given internally is sodium salicylate.—*Med. and Surg. Reporter.*

